# Robbery and kidnapping xperiences among bank employees: narratives of exposure and the aftercare received

**DOI:** 10.47626/1679-4435-2022-1039

**Published:** 2024-09-24

**Authors:** Manoel Juvenal da Costa Neto, Sergio Roberto de Lucca, Claudio José dos Santos Júnior, John Victor dos Santos Silva, Juliane Cabral Silva, Mara Cristina Ribeiro

**Affiliations:** 1 Centro Universitário Cesmac, Maceió, AL, Brazil; 2 Universidade Estadual de Campinas, Campinas, SP, Brazil; 3 Universidade de São Paulo, São Paulo, SP, Brazil; 4 Universidade Estadual de Ciências da Saúde de Alagoas, Maceió, AL, Brazil

**Keywords:** exposure to violence, stress disorders, post-traumatic, violence, occupational health, mental health, exposição à violência, transtorno de estresse pós-traumático, violência, saúde do trabalhador, saúde mental

## Abstract

**Introduction:**

Increasing urban violence exposes bank workers to traumatic situations in the work environment.

**Objectives:**

This study investigated the perceptions of bank workers about their robbery and/or kidnapping experiences and the aftercare they received.

**Methods:**

This qualitative study involved in-depth interviews with 7 workers from a public banking institution in 4 states in the northeastern region of Brazil who victims of kidnappings or robberies.

**Results:**

Their experiences were classified into 2 thematic categories: the dynamics of the event and the perception of aftercare actions.

**Conclusions:**

The violent experiences caused significant and lingering suffering and trauma among the participants. They reported that the aftercare was limited and needs improvement. Caring for bank workers who have been victims of assault and kidnapping can prevent or minimize their resultant psychosocial suffering.

## Abstract (pt)

**Introdução:**

O aumento da violência urbana expõe trabalhadores bancários a situações traumáticas no ambiente de trabalho.

**Objetivos:**

Neste trabalho, objetivamos conhecer as percepções de trabalhadores bancários sobre a experiência vivida em assaltos e/ou sequestros e sobre a assistência à saúde recebida após esta vivência.

**Métodos:**

Adotou-se a pesquisa qualitativa, desenvolvida com sete trabalhadores(as) bancários(as) de um banco público, em quatro estados do nordeste brasileiro, vítimas de sequestros ou assaltos. A técnica utilizada para a produção dos dados foi a de entrevistas em profundidade.

**Resultados:**

As experiências narradas organizaram-se em duas categorias temáticas: dinâmica do evento e percepção das ações de cuidado após o evento.

**Conclusões:**

Em síntese, o estudo permitiu compreender que a violência experimentada fez emergir entre os participantes sofrimentos e traumas significativos que vão além do momento do evento. Em função disso, as narrativas indicaram que a assistência recebida após o evento traumático é limitada e poderia ser aprimorada. Assim, acolher e assistir os trabalhadores bancários vítimas de assalto e sequestro pode prevenir ou minimizar sofrimentos psicossociais advindos da vivência de violência.

## INTRODUCTION

The impact of urban violence has shaped the mortality profile of workers in different professions, whether through increased homicides, robberies, suicides, traffic accidents, kidnappings, death from stray bullets, or through conflict with clients or even co-workers.^1^

The violence and lack of security are cause for concern among various classes of workers. This concern is even greater in the banking sector due to the frequent robberies and kidnappings of employees who can unlock the vault, which makes them feel insecure and unprotected in their work environment.^2^

According to the Fifth National Bank Attack Survey, which was conducted by the National Confederation of Financial Workers (Contraf-CUT) and the National Confederation of Security Guards, there were 1484 incidents in Brazil in 2013, divided into two types: armed robberies (including kidnappings), and burglary (including ATM attacks).^3^ In 2016, 2082 incidents were recorded,^4^ while in 2018, 3388 incidents against bank branches, post offices, and lottery outlets were reported,^5^ indicating a significant increase in violence against banks and, consequently, bank workers.

Studies have identified different types of violence perpetrated against these workers. Robberies and burglaries of bank branches, armored cars, security companies, and ATMs are classified by the Brazilian police as attacks on financial institutions.^6^ Extortion through kidnapping is one way robbers seek to reduce confrontation with the police.^7^

It should be pointed out that when such violence occurs, not only do the people who are directly involved suffer, but their colleagues and family members are also affected directly or indirectly. In bank employee kidnappings, it is quite common for families to also suffer direct violence as a strategic component of this type of crime.^8^

Frequent exposure to workplace trauma can result in work-related psychological disorders. A survey of

Italian banks^9^ concluded that employees at greater risk of post-traumatic stress disorder (PTSD) after a robbery can be identified by the intensity of their immediate reactions. The same study found that bank managers should pay greater attention to employees who have been previously victimized, since they are more vulnerable to mental illness. Moreover, during interventions, worker perception of the threat must be considered, rather than focusing solely on objective damage.^9^

Despite the growth of urban violence and frequent attacks on bank workers, few studies have been conducted on the subject. Data on the impact of this violence on worker health, especially mental health, is still limited and does not encompass the range of suffering and mental illness that these experiences have caused.

This study analyzed the perception of bank workers about their robbery and/or kidnapping experiences and the aftercare they received from their employers.

## METHODS

This qualitative exploratory study prioritized the victims’ narratives, including the processes they underwent and the meaning they attributed to these experiences.^10^ The participants worked in branches of a single public bank in the states of Alagoas, Bahia, Pernambuco and Sergipe and experienced an armed robbery and/or kidnapping pursuant to their duties in the last 6 years.

According to the bank’s security department (which listed occurrences), and its human resources department (which provided data on exposed workers), in the last 6 years, 7 employees were kidnapped and 143 were involved in robberies. Ten employees were involved in 2 incidents, totaling 150 exposures among 140 workers. Six of these workers were excluded due to death, contract termination, or transfer to a different state. The remaining 134 employees were invited directly or by email to participate in the study, of whom 37 (27.6%) were initially willing to participate. However, due to subsequent withdrawals, only 7 participated in in-depth interviews.

Data were produced through in-depth interviews, since this approach allows participants to speak freely about the subject. The intent of the interviewer’s questions was to lead to deeper reflection on and a clearer understanding of the respondents’ statements. From this perspective, the dialog between interviewer and interviewee was expanded into a privileged space in which the respondents’ opinions, experiences, and emotions could be expressed, with the interviewer guiding the interaction.^11^

The interviews were carried out by a single researcher according to a script guided by the following questions: “When, where, and how did the robbery and/or kidnapping in which you were involved take place?”; “Have you ever experienced any previous psychological trauma (assault, kidnapping, violence, abuse, etc.)?”; and “I would like you to tell me, in detail, what health actions you received from the company after being kidnapped/robbed and your assessment of them”.

With participant permission, the interviews were recorded on a cell phone and then manually transcribed in full for in-depth analysis. To help ensure anonymity and the confidentiality of information, the participants will be identified by fictitious names.

The narratives were interpreted using the content analysis technique, which involved a free-floating reading of the transcribed narratives, through which the core meanings behind statements are discovered, resulting in the identification of thematic matrices and semantic categories.^12^ Thus, the discourse was analyzed by determining themes, organizing them into categories, and interpreting them in light of theoretical references.^13^

This study was approved by the institutional research ethics committee (decision 2,631,853; certificate 85961118.3.0000.0039).

## RESULTS AND DISCUSSION

Of the 7 interviewees, 4 were women (3 kidnappings, 1 armed robbery) and 3 were men (all armed robbery). Three participants worked in Alagoas, 2 in Pernambuco, 1 in Bahia, and 1 in Sergipe.

The data from the in-depth interviews were classified in 2 thematic axes: event dynamics and perceptions about aftercare

### EVENT DYNAMICS

The dynamics of the traumatic event were a relevant topic in the interviews. The narratives highlighted the employees’ perception of the criminals’ actions and the employees’ reaction to them, including the trauma they experienced.

#### Perception of the criminals’ actions

Bank robberies are characterized by quick, forced, and/or violent actions, in which firearms are used (or their use is threatened) to obtain victim cooperation.^7^ “*They arrived shooting at the branch’s door. Two were already inside waiting for the others to arrive. When the others fired and came in announcing the robbery, these 2 went and captured the guards. They went to the tellers, took the money and left. [There were] at least* 8” (Marcos). “One *kept putting the gun to my head and the other put the gun in my mouth and said ‘If you deny it one more time, you’re going to get shot’”* (João).

This description indicates that the violence is extreme in some cases, especially considering that what is at risk for victims is not being used to obtain the money or the password to the vault, but rather their very lives.

Some workers became emotional during the interview, indicating lingering and sometimes vivid trauma in their memory. However, in other reports, the violence was veiled, i.e., from the worker’s perspective, the criminals, even when facing death at the hands of police, remain calm. “*To be honest, they were very calm and polite. I don’t know if it was because we were women. They were never aggressive”* (Maria).

Two types of bank robberies were described. The first involves a large number of men, vehicles, and weapons, and the robbery is quick. In the second, the dynamics are more discreet; bank employees, mainly managers and treasurers and their families are kidnapped to obtain access to the vault.^6,7^

Worker kidnappings are usually planned well in advance. Weeks before the incident, the day-to-day lives of bank employees with access to passwords (the manager and treasurer) are studied in detail, including arrival and departure times and personal and confidential information about their private lives, place of residence, family composition, and habits.^6,7^ “In *the car, he said that he spent 1 month observing me. He knew where I lived, my clothes, my bag, everything. He knew where I did Pilates, work hours”* (Maria).

It is noteworthy that, despite the robbers’ calmness and politeness to Maria during the robbery, the impact of learning the quantity of information they collected about her daily life led to lingering feelings of vulnerability. This experience led the victim to understand that her life and that of her family was in constant risk. Thus, the intimidation in these situations goes beyond work, since criminals investigate the entire lives and habits of victims and their families, using this knowledge to terrorize them.^14^

*“They called us by name. They divided us. Each one in a car and headed towards the bank to carry out the robbery. And all the time threatening, threatening... saying that they had our family, showing photos, and that if anything went wrong, they would kill them. And if they didn’t kill us at that point, they would have us killed from prison. They said they had my family, that if I didn’t cooperate they were going to kill my mother. And before killing her (they) they were going to say that she was dying because of her daughter, and they were going to burn her alive and from prison they were going to have me killed”* (Lis).

Intimidation was reported as a traumatic experience, highlighting the victims’ fear, not only for their own life but of family members and co-workers. “*When I started crying, they aggressively told me to shut up and said: ‘you have to get to the bank and make it look like a normal day, you can’t cry’. He said:* ‘[If] *your friend doesn’t want to collaborate, we’re going to kill everyone’ and he kept saying he was going to kill us’”* (Lis).

The reports show that the experience caused a deep fear of a death, which, at the moment, seemed imminent. There is often a fear of physical aggression, as well, which could only be a threat or could actually happen.

#### Employee reaction

When faced with potentially traumatic situations, a range of reactions can result. Most distressing reactions do not fade, lasting weeks or months, and the employees’ statements show that people react to violent situations in different ways. “*I was very cold on the day. I told my colleagues to put their hands on their heads. I even warned them: ‘Guys, look down and put your hands on your head’”* (João).

The victims’ response to violence may be closely related to the strategies they resort to in daily life to reduce conflict and solve problems. João was concerned about the bank manager’s clinical condition and vulnerability, and decided to take responsibility and lead the criminals to the vault himself. “*The manager had recently returned. I believe it hadn’t been 2 months since she came back from a leave of absence due to depression. At the time, I think I was too cold. When I looked at her and saw she was crying, I couldn’t say: ‘Look, she’s the manager’! Right? I said: ‘Let’s go get the money’”* (João).

The conflict of whether to put oneself or others at risk, as reported by João, further aggravates the experience, and was reported by several employees. Maria’s example illustrates the ambiguous feeling experienced in moments of psychological pressure due to the robbers’ demands and uncertainty about the future. These feelings are increased through the suffering of other victims, causing feelings of humiliation, helplessness, and guilt.^7^ “*I can’t risk the lives of 3 people who are there because of money. I only thought about Clara’s mother and father, because I used to live with her in the country. So, I only thought about her mother. What would I say to her mother if something happened?*” (Maria).

Maria reports that time passed in slow motion: “*I sat there. The impression I had was that the world was in slow motion. From the time I decided to call until the police arrived, with everyone there, I was sitting in the chair and doing nothing. It seemed like everything was in slow motion. There was no confusion – it was like that, everything in slow motion.”* João also discussed the perceived dilation of time: “He *started shouting obscenities and saying he wanted it quickly, quickly. And that, like, was a very quick interval, but in the person’s head those seconds seem like an eternity...”*

These statements demonstrate that the perception of the experience is singular and, thus, charged with meaning. Responses during and after the event are also individualized, showing that the victims’ reactions are closely linked to their ways of understanding the experience. It should be pointed out that robbery and kidnapping victims normally report the experience as irreversible and permanent.^7^

An experience of this type can trigger mental suffering and the emergence of psychosocial risk and psychological disorders, including PTSD.^15^

João reported the following:

*“I spent approximately 7 days with that feeling from the gun. I kept asking my family if I had been injured and about the robber’s face, unable to sleep, insomnia. I started sleeping with the door to my room locked, which I had never done before, and I woke up at night with that (sigh), having some kind of nightmare, and I kept going from my room to the front door, feeling that someone was trying to break in my house, right? Then, 2 hours later, 3 hours later, I woke up feeling bad and went through it all over again.”*

Although not all robbery victims develop PTSD, they may present isolated symptoms of the condition over time, especially if the situation recurs, making them vulnerable to mental disorders.^16^ João was diagnosed with PTSD and took a leave of absence from work, using social security benefits. The fact that the victim is close to the aggressor and sees a firearm may be associated with an increased likelihood of adverse symptoms and a greater risk of PTSD, since the weapon represents a threat to the victim’s life.^16^

Furthermore, the way individuals react during or immediately after a traumatic situation appears to be more important regarding PTSD symptoms than the number of traumatic experiences.^17^

In Clara’s account, a previous mugging was more traumatizing than her kidnapping experience. “*I got mugged one time. They stole a necklace of mine. It was very dark. At the time, I couldn’t have been more traumatized. That was even more traumatic* [than the kidnapping]. *Because of the way they did it; because there was physical violence. He was a kid on drugs”* (Clara).

### PERCEPTION ABOUT AFTERCARE

The purpose of corporate occupational health services is to prevent illness among employees, especially work-related illness. Branch employees pass on information about the robbery or kidnapping to the bank security and human resources department, who confer with team members and contact the branch to verify the details and determine the necessary action, including referral to qualified care providers, such as psychologists and physicians.

After robberies or kidnappings, workers are provided with collective and individualized psychological assistance, a mode of immediate intervention used by several banking institutions.^18^ During collective care, screening is performed for the most affected workers so that they can be directed to individualized psychological and medical care.

#### Psychological care

The employees’ reports about psychological care indicate that different methods were used, such as a single session, collective therapy, and telephone support. “[The evaluation] *was good. She did a collective* [session], *with everyone, and then did it case by case, including the security guards, receptionists, and telephone operators”* (Marcos).

*“They took me to the break room, gave me sugar water, and then the psychologist called me. It was good. So, she talked to me, asked how I was. I don’t remember exactly, but I remember that she spoke to me, she talked. She asked if I was okay, if I was calm, if I had been medicated. I don’t remember much. I remember she spoke to me”* (Maria).

A meta-analysis of 12 randomized clinical trials involving a single session of psychological intervention found that, rather than a positive effect, it actually had a negative effect on patients who experienced more intense trauma. Group psychological interventions showed neutral results.^19^

Given the lack of scientific evidence regarding the effectiveness of specific interventions, researchers indicate the need for more complex interventions for individuals who present symptoms, such as trauma-focused therapy. This approach, which involves 4 to 12 sessions, has shown positive results for patients with PTSD.^19^

Despite the different approaches, the employees’ perception of the psychological care they received was, in general, positive: “I *found the service very good. I had no idea there would be a psychologist to talk to us right then. Mainly because he said that if we wanted, we could even have a follow-up later”* (Clara).

Despite these positive reports, the literature indicates the importance of early intervention and psychosocial support, given that inadequate care provision is associated with developing PTSD, and delays in treatment can delay recovery.

“[The kidnapping] *was on a Friday. On Saturday morning, I already told the bank managers ‘I need help because I can’t sleep, I’m just crying’. I went to an emergency room, took a tranquilizer. On Sunday afternoon, I went* [to see the psychologist]. *I had about 3 emergency sessions and then I looked for* [another] *psychiatrist or psychologist for* [further] *therapy* [...] *I think it was positive”* (Lis).

It should be pointed out that mobilizing company resources to prevent work-related mental suffering is more effective than using the public health system.^20^ It has also been reported that pressuring victims to talk about the trauma moments after it has occurred can delay recovery and reinforce feelings of helplessness. However, there are cases in which trauma patients are able to talk about what happened with family, friends and health care professionals.^21^ Thus, workers should be adequately assessed and referred for specialized treatment immediately after the event.^22^ They should be made to feel at ease to talk or not talk about the event. However, they should be given all the time they need to seek help, not just during the immediate aftermath of the event.

#### Medical care

According to the employees’ statements, medical care is not always provided and, when it is, it does not always meet expectations. Of the 7 interviewed bank employees, only two received medical care from the company. Clara highlighted the importance of this support and how not receiving it affected her: “It *was lacking. Someone could have been there, preventively, since it was unknown whether it would be needed. Having someone there, in case we needed something, had a blood pressure spike, got very upset, or needed medication”.* Thus, Clara was clearly concerned about the immediate health care that could be provided.

It should be considered, however, that the role of occupational physicians goes beyond immediate care. The skills required to practice occupational medicine include the ability to assess, analyze, and intervene in situations involving risk to the physical or mental health of workers. The professional must initiate changes in the care process and develop strategies to protect worker health.^23^ Occupational health services must also identify individuals at risk, advise management about needed changes in the workplace, train supervisors to identify late manifestations of PTSD, train workers about resilience strategies and healthy behaviors, provide health services, maintain a multidisciplinary team with the skills to provide appropriate treatment, monitor and screen employees for mental health risk, monitor workers at risk of re-exposure, and provide accessible treatment and psychological assessment when there are nonspecific physical symptoms.^24^

However, this does not always happen in practice. According to João: “Dr. [name withheld] *said ‘How are you all? Blah, blah, that’s it. Everyone just asked everyone how they were and they immediately said ‘I’m fine, I’m fine’. OK. You can go. That was the post-robbery care. The doctor also made himself (available), but this was only the care we received at first”* Based on this account, it is apparent that the care he received, both in the peri- and post-traumatic periods, was flawed. He received no immediate care from the company, and the psychological risk from his trauma was not subsequently considered. According to João, he was not allowed a leave of absence and was kept in the same work environment in which the trauma occurred, despite showing no signs of readiness to return to work.

To manage psychosocial risk in the workplace, especially when it is related to violence, 3 levels of intervention have been suggested: (1) primary interventions, which include reporting events and designing work environments free of psychosocial risk; (2) secondary interventions, also called timely reactions, which aim to increase individual resources and provide violence-related interventions, training, research, and case resolution; and (3) tertiary interventions, which involve business agreements and aftercare, in addition to counseling and therapy programs, to reduce and resolve the harm caused by violence.^25^

Based on their reports, many participants expressed a desire for closer monitoring by the occupational physician, and not just immediately after the event. The need to think about changing the branch’s location was even mentioned, since there were reports of psychological suffering from returning to the scene of the crime.

#### Attention from company managers

The narratives also show that the attention management pays to employees who have been exposed to violence also has its effects. “*Even the general manager came. I can’t question that. He came, talked to everyone, asked if everyone was well and able to work and whether anyone wanted to go home. I can’t complain about that”* (Jorge). Although such attention is not health care, it can have positive and protective effects against psychosocial suffering and even PTSD, since it expresses the company’s social support.^26^ However, such gestures do not always occur. The greatest concern of managers is often keeping the branch open for business, which can worsen the situation for employees, as in the following reports:

*“The [management representative] responded like this: ‘How are you? Are you well?’ [...] Then she proposed [... ] ‘it would be good if you could go to the branch to calm the team down’. She asked: ‘Did something happen to anyone else?’ I said ‘No, just me and him’. She said: ‘Well, in these situations the team becomes very scared. Maybe they won’t want to go to work tomorrow’”* (João).

Some employees reported that the managers cared more about preserving the company’s resources and keeping the branch open than the workers’ mental health. “*Some time later there was a meeting at headquarters and a manager stated in that meeting, that the most important thing was to maintain the company’s financial resources, showing no concern for the employees.”* (Maria).

Since caring for workers is, first and foremost, an ethical decision to preserve their health, it should not be restricted to interventions by health professionals. To deal with such incidents, the banking sector should develop a crisis prevention and management plan. This includes everything from agency security, with staff trained to prevent these occurrences, to measures guaranteeing adequate support for traumatized workers, so that they can return to the work environment in safety and health.

In cases of robbery or kidnapping, immediate post-event interventions are of fundamental importance. Medical health monitoring is always recommended, but mandatory psychological evaluation is not recommended.^20^ Instead, a longitudinal monitoring program should make psychological or psychiatric support available when recommended by the occupational medicine department. Therefore, management must provide a multidisciplinary team to care for workers who have experienced violence at work. Some important aspects for better care outcomes after an event might include: having a team trained to identify individuals at risk of psychosocial disorders, considering changing the branch’s location, if necessary, and courses, training, or workshops on stress reduction strategies for workers.

### STUDY LIMITATIONS

Several study limitations should be highlighted, the first of which is that the results are based on the experiences of employees of a single public banking institution in the northeastern region of the country. The results may vary in other regions and in private banks. The qualitative design, involving in-depth interviews, proved to appropriate for the topic, although the convenience sampling methodology prevents correlations or generalizations regarding other public or private banks. This topic should be explored in greater depth with other methodologies and in different banking institutions and regions. This will be essential for a clearer understanding of the phenomenon and to determine the changes needed in care for employees who are exposed on a daily basis to urban violence, specifically, robberies and kidnappings.

## CONCLUSIONS

This study showed that bank robbery and kidnapping events caused significant and lingering trauma to the victims. Their narratives indicate that the aftercare they received was limited and needs improvement. Banking institutions should recognize the psychosocial risks to which their workers are exposed and the psychological harm these events can cause, including negative repercussions for their families, other employees, and society in general. Banks must train their crisis management teams to provide the necessary support to victimized workers. Post-trauma care protocols must be created that allow workers to receive continuing psychological care, even in the absence of complaints or symptoms, in addition to medical assistance. When necessary, long-term care must be provided without creating additional pressure on the day-to-day work routine, i.e., respecting employee autonomy regarding whether or not to receive treatment.
